# An Intron Mutation in the *ACVRL1* May Be Associated with a Transcriptional Regulation Defect in a Chinese Family with Hereditary Hemorrhagic Telangiectasia

**DOI:** 10.1371/journal.pone.0058031

**Published:** 2013-02-27

**Authors:** Qian Yu, Xiao-hui Shen, Ying Li, Rui-juan Li, Ji Li, Yun-ya Luo, Su-fang Liu, Ming-yang Deng, Min-fei Pei, Guang-sen Zhang

**Affiliations:** Division of Hematology, Institution of molecular hematology, The Second Xiang-Ya Hospital, Central South University, Changsha, Hunan, People’s Republic of China; University of Bonn, Institut of Experimental Hematology and Transfusion Medicine, Germany

## Abstract

**Purpose:**

To identify a novel pathogenic gene mutation present in a Chinese family with hereditary hemorrhagic telangiectasia (HHT) and to determine if an intron mutation may influence the transcriptional activity of the *ACVRL1* gene.

**Methods:**

HHT family members were ascertained following the presentation of proband and involved subjects. All family members (n = 5) and 113 healthy individuals were genotyped for the variant in intron 6 c.772+27G>C of *ACVRL1* gene. The genomic structure of *ACVRL1* in affected HHT patients and healthy individuals was determined by long range PCR and sequencing. The expression of *ACVRL1* mRNA and protein in patients with HHT was evaluated using real-time polymerase chain reaction and immunoblot analysis. Luciferase activity assay and electrophoretic mobility shift assay (EMSA) were performed to uncover the mechanism of intron-related transcriptional regulation.

**Results:**

Only one novel mutation in intron 6 (c.772+27G>C) of *ACVRL1* gene, no other mutation, abnormal splice, gross genomic deletion or rearrangement was found in this HHT2 family. Compared with healthy individuals, *ACVRL1* mRNA and protein were significantly decreased in affected HHT2 individuals. Luciferase activity assay demonstrated that the transcriptional activity of the mutated *ACVRL1* was significantly lower than that of the wild-type of intron 6; EMSA results showed that intron 6 c.772+27G>C mutation was able to inhibit the binding of transcriptional factor Sp1.

**Conclusions:**

A novel intron mutation in *ACVRL1* gene is associated with familial HHT2. The mechanisms may be involved in the down-regulation of *ACVRL1* gene transcription.

## Introduction

Hereditary hemorrhagic telangiectasia (HHT) is an autosomal dominant disorder, characterized by multi-system vascular dysplasia such as mucocutaneous telangiectases, and arteriovenous malformations (AVMs) in the lung, liver and brain. Telangiectases may lead to epistaxis and gastrointestinal (GI) bleeding. Patients with HHT are at risk of developing arterial to venous shunts that may cause serious complications including stroke and other types of hemorrhage [1], [2], [3]. Most of the published families with HHT fall into two groups: HHT1, mapping to chromosome 9q34.1 [4]; and HHT2, mapping to chromosome 12q13 [5]. There are subtle differences in the phenotype between HHT1 and HHT2, with HHT2 patients exhibiting fewer pulmonary AVMs and a milder HHT phenotype [6]. A higher risk for lung involvement has been suggested in HHT1 [1], while in some HHT2 families, GI bleeding and problems in the liver are more predisposed [6], [7], [8]. However, direct evidence for GI telangiectases is scarce.

HHT1 has been linked to mutations in the endoglin (*ENG*) gene [9], whereas HHT2 has been linked to mutations in the activin receptor-like kinase-1 (*ACVRL1*) gene [2], [10]. However, screening for mutations in either the *ENG* or *ACVRL1* gene in patients with HHT yields rates of mutation detection of between 62% and 93% [10], [11], [12], implying that most DNA scans for mutations share common detection limitations. A subset of the mutation negative samples may instead have a mutation in which the locus is linkaged to 5q31.3–32, and has recently been suggested to be the *SMAD4* gene, which was identified as being mutated in a subset of patients with HHT3 who also suffer from juvenile polyposis (JP). Therefore, the condition also is simply referred to as JP/HHT [13], [14], [15]. The *SMAD4* protein is a key downstream effector of transforming growth factor β (TGF-β) signaling. In 2006, Gallione *et al.* reported that the incidence of *SMAD4* mutation was 10% in patients with HHT who were negative for mutations in either *ENG* or *ACVRL1* [16]. With this fact as basis, the authors propose that routine DNA-based screening for HHT should include a test for *SMAD4* mutations in samples where neither *ENG* nor *ACVRL1* mutations are identified.

Because approximately 10% of clinically diagnosed patients with HHT have unidentified mutations, the study of intronic sequences, splice sites, and promoter regions of both the *ACVRL1* and *ENG* genes is critical importance. Moreover, haploinsufficiency is currently accepted as the basis for the pathogenesis of HHT [17], [18], and therefore, an understanding of the mechanism on gene transcription may be crucial to identify strategies to counteract haploinsufficiency. However, few data relating to deep intronic mutations in *ACVRL1* have been reported. We report here an intronic mutation in two members of a Chinese family who meet clinical diagnostic criteria for HHT [18]. After completing DNA sequencing on all coding regions in the *ACVRL1*, *ENG*, and *SMAD4* genes, and the genomic structure assay for *ACVRL1* gene, a novel mutation in intron 6 of *ACVRL1*, c.772+27G>C, was identified in this family. In addition, we used real-time polymerase chain reaction (PCR) and immunoblot analysis to demonstrate the downregulation of the expression of both *ACVRL1* mRNA and protein in the affected family members. We further characterized the functional activities of the point mutation in intron 6 and concluded that this mutation may be involved in the defective transcriptional regulation of *ACVRL1*.

## Materials and Methods

### Subjects

**Figure 1:**
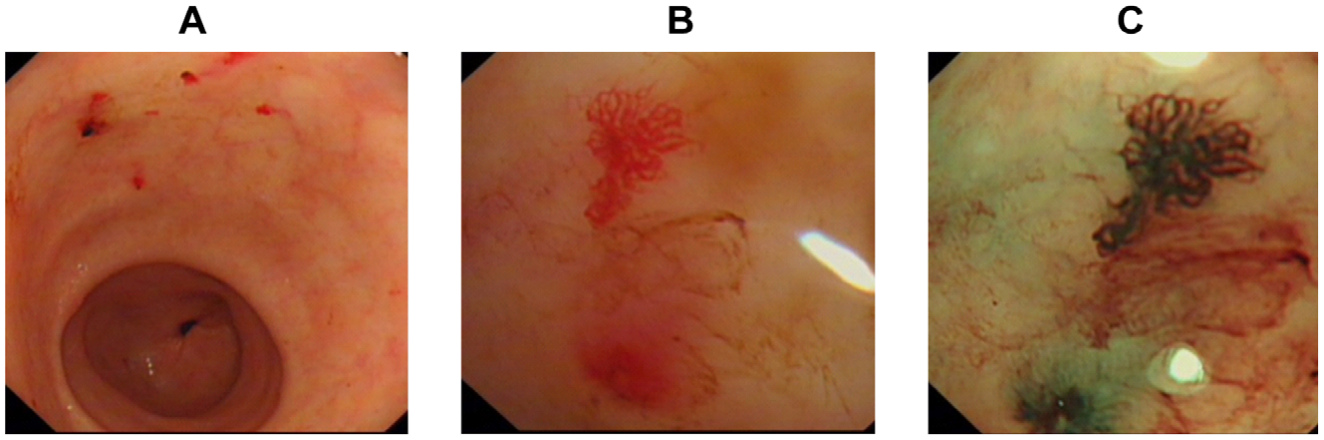
Magnification endoscopy with narrow band imaging (NBI) pictures of the proband’s gastric mucosa. A. Example of endoscopy images showing four red spots, indicating the presence of telangiectasia of the mucosa with slight oozing of blood. B. A magnified view of a single spot on the mucosa. C. NBI of the spot shown in B.

The proband, a 52-year-old woman, was referred to the Second Xiang-Ya Hospital for recurrent epistaxis, intermittent black stools, and severe anemia, which had occurred for more than 35 years. With informed consent, an extended pedigree analysis was performed. The proband’s son, a 25-year-old young man had had recurrent epistaxis since age 10. Both the proband and her son had evidence of telangiectasia in their noses, lips, tongues, and the mucosa of their oral cavities. A third member of the family, the proband’s 2-year-old grandson, had occasional epistaxis, but no mucocutaneous telangiectasia was found. None of the remaining members of the family complained of bleeding or telangiectasia. Liver ultrasound and pulmonary CT scans did not reveal any arteriovenous malformation (AVMs) in the proband or her son. An upper GI endoscope examination in the proband showed that the gastric mucosa, including the gastric antrum, gastric body, and gastric angle, presented multiple erythema-like changes with slight oozing of the blood. The lesions were further confirmed to be telangiectasia by magnification endoscopy with narrow band imaging (NBI) technique (Fig. 1). The diagnosis of HHT for the proband and her son was made by the presence of four international consensus diagnostic criteria [18], that is: affected first degree relative, recurrent spontaneous nosebleeds, mucocutaneous telangiectases, and documented visceral manifestations. According to the proposal, the proband presented the four suggested diagnostic criteria (including gastric mucosa telangiectasia), and her son meet the three diagnostic criteria (a positive family history; recurrent epistaxis and lips, tongue, oral mucosa telangiectases).

### Ethics Statement

All samples were collected from study participants after obtaining written informed consent under clinical research protocols approved by the institutional review board of the Second Xiang-Ya Hospital, Central South University. The written informed consent of the proband’s grandson was obtained from his parents.

### Sample Preparation, Genetic Screening and Localization of *ACVRL1* Introns

Genomic DNA was isolated from the peripheral blood mononuclear cells (PBMCs). All 5 members of the family were screened for mutations in the *ACVRL1*, *ENG* and *SMAD4* genes by direct DNA sequencing of PCR products that included all coding exons. Any variations in the sequence that were detected were re-amplified and sequenced again to confirm the observed findings. The sequence results were compared with wild-type *ACVRL1* (GeneBank, NT_029419.11), *ENG* (GeneBank, NT_008470.18) and *SMAD4* (GeneBank, NT_010966.13). In order to exclude all rearrangement that could be involved in the *ACVRL1* gene and confirm genomic integrity, all introns for the *ACVRL1* gene were amplified by long range PCR on proband, proband’s son and one healthy individual, and sequences were performed. All primers are shown in table 1. A total of 113 normal individuals were screened for the c.772+27G>C mutation of intron 6 in the *ACVRL1* gene using direct DNA sequencing (20 individuals) or a StuI restriction endonuclease assay (93 individuals), where the G to C substitution predicts the creation of a single StuI site in the intron 6 amplified products.

**Table 1:** Primers for amplification of introns of *ACVRL1*.

#	Primer (5′-3′)	Genomic position of theprimers in *ACVRL1*(chromosome 12q13)	Annealingtemperature(°C)	Size(bp)	Exon*
1	sense: AGCAAAGGCTGCCAAGGA	3239–3256	60.0	2394	2
	antisense: CTGAGCCCGACACCCATT	5632–5615			
2	sense: GGGAGCAGTTAGGAAACAGGA	5130–5150	59.0	2750	3–7
	antisense: GCTCGTGGTAGTGCGTGATG	7879–7860			
3	sense: GTAGAGACGAGGTCTCCCTATGT	7508–7530	60.0	2413	7–8
	antisense: GCAACCCACAGTAACAACCAG	9920–9900			
4	sense: TGACCGTGCGTAGGTTATTTGAC	9223–9245	60.0	2396	9
	antisense: GGTCATTGGGCACCACATCAT	11618–11598			
5	sense: CCACAGGGCTGTTGTGAGGA	11302–11321	60.0	2497	9–10
	antisense: TTCCCTGGCTTGGCACTCT	13798–13780			
6	sense: TGATGCGGGAGTGCTGGTA	13364–13382	60.0	2775	10
	antisense: TCCCAATCTGGGGTAGGAACT	16138–16118			

* Exons amplified in each long range PCR product of *ACVRL1* gene.

### PCR Amplification of the *ACVRL1* cDNA

To confirm the integrity of the *ACVRL1* mRNA and to exclude abnormal splicing, overlapping RT-PCR was carried out on proband and her son. Total cellular RNA from 1×10^6^ cells/ml was extracted from peripheral blood mononuclear cells by Trizol reagents (Invitrogen). First-strand cDNA was synthesized by M-MLV reverse transcriptase using random primers (RT Kit, Fermentas). With the cDNA generated, primer pairs were designed to amplify each exon of the *ACVRL1* gene, and PCR products were identified by sequencing. All primers are shown in table 2.

**Table 2:** Primers for amplification of *ACVRL1* cDNA.

#	Primer (5′-3′)	Genomic position of theprimers in *ACVRL1*(chromosome 12q13)	Annealingtemperature(°C)	Size(bp)	Exon*
1	sense: TGTCACACTTCATGGCTCTTACTC	5005–5028	59.0	327	2–3
	antisense: TGTCGCAGCAGTAGTGGTTGA	5894–5874			
2	sense: CTTCATGGCTCTTACTCCACCTC	5012–5034	59.0	892	2–7
	antisense: GTGCTCGTGGTAGTGCGTGA	7881–7862			
3	sense: CGTCAACCACTACTGCTGCGA	5872–5892	60.0	1008	3–9
	antisense: TCCTCCACGATGCCATTCAC	11580–11568, 8816–8810			
4	sense: GATTACCTGGACATCGGCAACAAC	8648–8671	60.0	532	8–10
	antisense: CACCACACTCACACTACCTCTACC	13573–13550			

* Exons amplified in each RT-PCR product of *ACVRL1* gene.

### Real-time PCR for Evaluating mRNA Expression of *ACVRL1*, *ENG*, and *VEGF*

Real-time PCR was performed to evaluate the expression levels of *ACVRL1*, *ENG* and vessel endothelial growth factor (*VEGF*) genes, and to assess whether the mutation in intron 6 of *ACVRL1* gene interfered with the transcription of *ACVRL1* mRNA; and if *ENG* or *VEGF* mRNA expression was upregulated as a compensation mechanism. Blood samples (20 ml) from all 5 members of the family and 6 healthy individuals were included in the study. Based on the reported literatures, in which Lastres *et al.* had demonstrated that activated monocytes/macrophage cells are able to express endoglin and *ACVRL1* [19], [20], we chose PBMCs as research material. Briefly, PBMCs were isolated by Ficoll centrifugation, and the total cellular RNA was extracted using Trizol reagents (Invitrogen). The RNA was reverse-transcribed using A3500 Reverse Transcription System (Promega). Primer sequences were as follows: human *ACVRL1*-specific primers, 5′-TTGCTCAGACACGACAACATCC-3′ (sense) and 5′-ACATTGCGGCTCTTGAAGTCG-3′ (antisense); human *ENG*-specific primers, 5′-TATTGACCACCGCCTCATTGC-3′ (sense) and 5′-GCTGCTCATGTCCTTGATCC-3′ (antisense); human *VEGF*-specific primers, 5′-GAGCCTTGCCTTGCTGCTCTAC-3′ (sense) and 5′-CACCAGGGTCTCGATTGGATG-3′ (antisense); human *GAPDH* primers, 5′-AATCCCATCACCATCTTCC-3′ (sense) and 5′-CATCACGCCACAGTTTCC-3′ (antisense). Real-time PCR using SYBR Green I dye (BIOER Technology Co. Ltd., Hangzhou, China) was carried out on a Rotor-Gene 6500 HRM (Gene Co. Ltd., USA). Relative mRNA abundance was calculated using *GAPDH* as the internal control using the 2^−ΔΔCT^ method. For each experiment, mRNA levels from healthy individual were used for comparison and set to 1. The mRNA levels were represented relative to healthy individual.

### Immunoblot Analysis

Blood samples were taken from the family members with HHT and healthy individuals. PBMCs were prepared as described-above. Cells were lysed in a buffer containing 10 mM Tris-HCl (pH 9.0), 140 mM NaCl, 0.5 mM MgCl_2_, 1 mM CaCl_2_, 1 mM DTT, 0.5% NP-40, 1 mM phenylmethanesulfonyl fluoride (PMSF), and 2 µg/ml aprotinin for 30 min on ice. Equal amounts of protein (100 µg/well) were resolved on 8% sodium dodecyl sulfate–polyacrylamide gel electrophoresis (PAGE) and transferred to nitrocellulose membranes. The membranes were blocked for 1 hour in Blotto/Tween buffer (5% nonfat milk powder, 0.2% Tween 20 in PBS) and probed with primary antibodies: rabbit anti-human ACVRL1 antibody (Abcam, UK) (1∶500) and mouse anti-human actin antibody (Santa Cruz) (1∶1000), respectively. The protein signal was visualized using an ECL detection system (Amersham).

### Nuclear Extracts and Electrophoretic Mobility Shift Assay (EMSA)

**Figure 2:**
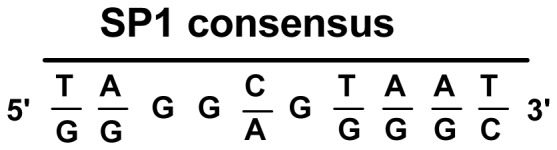
The consensus Sp1 binding site.

Nuclear extracts from PBMCs were prepared according to the instructions contained in a nuclear extract kit (Active Motif Inc, USA). The EMSA assay kit was purchased from Beyotime Institute of Biotechnology (Haimen, Jiangsu Province, China). The prediction of the transcriptional factor binding site for the *ACVRL1* intron 6 mutation was performed with AliBaba 2.1 software (www.gene-regulation.com/pub/programs.html), and the result suggested that the mutant sequence could bind to the Sp1 protein. The consensus Sp1 binding site is shown in Figure 2 [21]. Three double-stranded oligonucleotide probes, including a Sp1 general probe, an *ACVRL1* intron 6 wild-type probe that covered the +5–+34 bp region of intron 6, and an additional mutated probe (+5–+34 bp) containing the patient’s G to C mutation at position 27 in intron 6, were synthesized and labeled with biotin in Shanghai Biosune Biotechnology Co. Ltd. (Shanghai, China). EMSA was performed as previously described [22] with slight modification. The sequences of the probes were as follows: Sp1 general probe, 5′-attcgatcggggcggggcgagc-3′ (sense) and 5′-gctcgccccgccccgatcgaat-3′ (antisense); *ACVRL1* intron 6 wild-type probe, 5′-ggggagaggccagctgtgccaggcctgggg-3′ (sense) and 5′-ccccaggcctggcacagctggcctctcccc-3′ (antisense); mutant probe (+5–+34 bp) containing the patient’s G to C mutation at position 27 in intron 6, 5′-ggggagaggccagctgtgccagccctgggg-3′ (sense) and 5′-ccccagggctggcacagctggcctctcccc-3′ (antisense).

Binding reactions were conducted in a total of 10 µl volume with nuclease-free water, 5×EMSA/gel-shift buffer, 2 µg of nuclear extract, and the biotin-labeled probes. For the competitive reactions, unlabeled probe (in excess of 100 times) also was added and incubated for 20 min before the addition of labeled DNA. After incubation, reactive mixtures were separated using electrophoresis on 6% native PAGE at 100 V in 0.5×TBE buffer. Then, the DNA mixture was transferred onto nitrocellulose membranes, and the signal from the probe was detected using an ECL detection system (Amersham).

### Reporter Gene Construct and Luciferase Activity Assay

To further testify whether intron 6 c.772+27G>C mutation could affect the transcriptional activity of the *ACVRL1* gene, corresponding reporter gene constructs were created as follows: 1). the pGL3-promoter vector (empty vector, Promega, USA), containing the SV40 promoter of driving the firefly luciferase gene; 2). pGL3-pro-ACVRL1-int6-wt, containing the luciferase reporter gene and carrying 639 bp of *ACVRL1* intron 6 (wild type); 3). pGL3-pro-ACVRL1-int6-mt27 (mutant type), containing the intron 6 c.772+27G>C mutation. The intron 6 and its mutant form (+27G>C mutation) were respectively inserted into the junction area between exon 5 and exon 6 of luciferase gene. The constructions of the reporter gene were performed by TaKaRa Company using In-Fusion cloning technology (Dalian, China). Firstly, we prepared a linearized vector by NcoI/XbaI restriction digest; the linearized vector was purified and retrieved using TaKaRa MiniBEST agarose gel DNA extraction kit (Code No.D823A). Secondly, PCR amplification of target fragments was performed, including the intron 6 (primer A) of *ACVRL1* gene in the patient (int6-mt27) or in the healthy individual (int6-wt), and the luciferase gene in pGL3-promoter vector (luciferase B, covering exon 1–5; luciferase C, covering exon 6–7, respectively). Primer (A, B, C, D) details are available on request. Thirdly, we mixed PCR productions together (including int6-wt+ luciferase B+ luciferase C; and int6-mt27+ luciferase B+ luciferase C, respectively) and performed the second round PCR amplification with primer D. The primer D shares 15 bases of homology with the ends of the linearized vector. After PCR amplification, the recombination target fragments (including luciferase B+ int6-wt+ luciferase C and luciferase B+ int6-mt27+ luciferase C) were generated. Finally, In-Fusion cloning was finished according to the instruction of In-Fusion® HD cloning kit (TaKaRa Clontech Code No. 639648). Briefly, for fusion cloning, the reaction system included: 5× In-Fusion HD enzyme premix 2 µl, linearized vector 3 µl, purified PCR fragment 1 µl, adjusted the total reaction volume to 10 µl using ddH_2_O and the reaction mixtures were incubated at 50°C for 15 minutes. After that, the target fragments and linearized vector ends were ligated and fused by the 15 bp of homologous sequence derived from the ends of the linearized vector. The clones derived from transformation were picked up randomly and identified by regular procedures.

All constructs were sequenced to define their size and ensure the completeness of the constructs. A PCR-based strategy was applied to introduce the mutated or wild-type DNA from the patient or healthy individual into plasmid vectors. The cloned PCR fragment was sequenced for excluding potential PCR-induced sequence errors.

Human umbilical vein endothelial cell line (HUVECs: CRL-1730) was from ATCC [23]. The cells were maintained as nonsuspension cultures in RPMI-1640 media, and supplemented with FBS and antibiotics. To perform the luciferase activity assay, 5×10^5^ HUVECs grown in 90-mm culture plates were co-transfected with 4 µg of firefly luciferase reporter vectors, containing the wild-type, mutant construct or empty vector, and 0.4 µg of the internal control plasmid pRL-TK using 12 µl Lipofectamine™ 2000 (Invitrogen, USA) as described in the instruction. The relative luciferase activity was normalized over the TK activity value. At 6 h after transfection, RPMI-1640 with FBS and antibiotics was added into HUVECs. 48 hours later, the cells were washed twice with 1×PBS, suspended in 500 µl reporter lysis buffer, LARII and Stop&Glo™ buffers were added into the reporter lysis buffer, respectively. The measurement of the firefly and renilla luciferase activities was performed by Dual-Luciferase reporter assay system (Promega, USA) with a GloMax 20/20 luminometer (Promega, USA). The experiments were repeated at least three times. All results were expressed as fold increases or decreases over the level produced by cell transfection with the pGL3-promoter vector (control vector). In addition, we constructed another report gene system, where both wild-type and mutant-type intron 6 were inserted into the upstream of the promoter-luc^+^ transcriptional unit of the pGL3-promoter vector, and the luciferase activities assay was finished as mentioned above.

## Results

### Identification of *ACVRL1* Mutation

**Figure 3:**
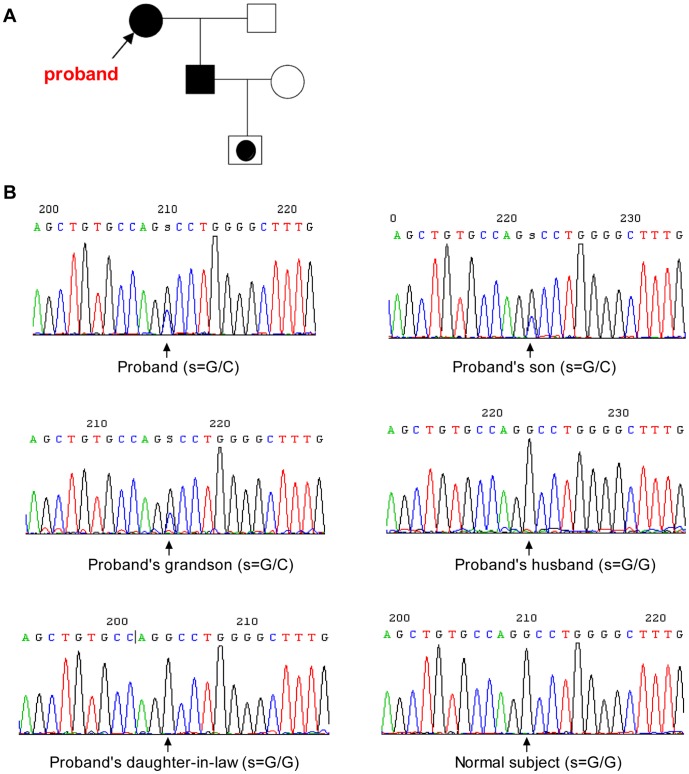
Pedigree and novel mutation in intron 6 c.772+27G>C of *ACVRL1* gene in the HHT family. A. Pedigree: Male (▪) and female (•) family members are shown. The filled symbols indicate patients with HHT. Dotted square indicates patient with possible HHT. B. Results of DNA sequencing of intron 6 from all members in the family and healthy individual.

The pedigree of the family is shown in Figure 3A. The DNA of the proband and her family members was successfully amplified, and the PCR products were directly sequenced. No mutations were found in either the coding exons or adjacent intronic regions of *ENG* and *SMAD4* genes in the proband and her affected son. However, the proband, her son, and her grandson all shared the same mutation at position c.772+27G>C in intron 6 of the *ACVRL1* gene (Fig. 3B), implying the presence of a heterozygous mutation. No other abnormalities were identified in any other regions of the *ACVRL1* gene, including all exons. In addition, the c.772+27G>C mutation in intron 6 of the *ACVRL1* gene was not present in the proband’s husband or daughter-in-law. In normal controls (*n* = 20) where PCR products were directly sequenced, the mutation was negative. Because the G to C substitution predicted the creation of a new StuI restriction endonuclease site, the sequencing results were further confirmed using restriction fragment length polymorphism (RFLP) analysis of DNA from the family members. The StuI RFLP results showed that the DNA of the proband was cleaved into three fragments (294 bp; 256 bp; and 38 bp) by StuI digestion. The DNA of the proband’s son and grandson presented identical changes. However, with DNA from the proband’s husband and her daughter-in-law, only two bands (256 bp; 38 bp) were visible, which were identical to those of RFLP from healthy individuals (*n* = 93). Therefore, the mutation at position c.772+27G>C in intron 6 of the *ACVRL1* gene was absent in a total of 113 healthy individuals, suggesting that the mutation is not a polymorphism.

### Genomic Structure of *ACVRL1* in Involved HHT Patients

**Figure 4:**
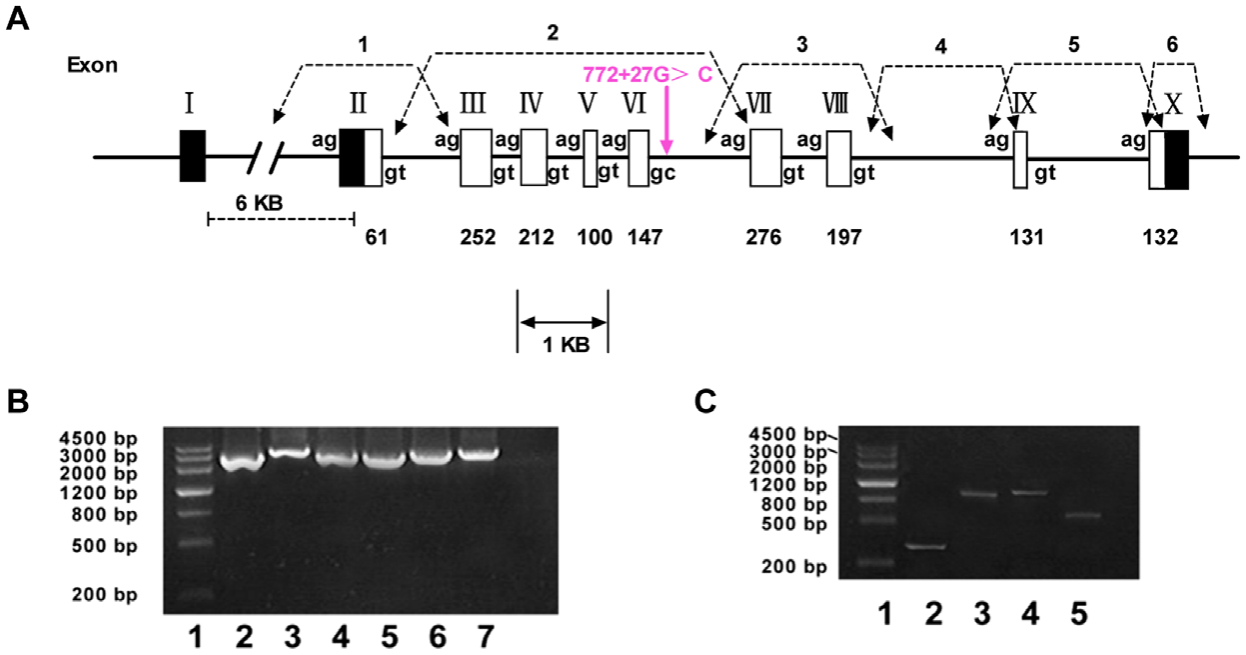
Long range PCRs and RT-PCR of *ACVRL1* gene. A. Schematic diagram of the *ACVRL1* gene, determined by long range PCR and sequencing. The exons are marked by boxes unblackened for the coding regions and blackened for the noncoding regions. Exact exon size (in bp) is given below the exon. The dotted line is indicated for the putative alternative splice, in the 5′UTR and all introns follow the GT–AG rule except for intron 6. The novel mutation point: intron 6 c.772+27G>C in the present family is marked using purple arrow. The curved arrow marks the borders of each long range PCR product of *ACVRL1* gene. Primers details in table 1 B. Agarose gel electrophoresis of *ACVRL1* gene long range PCR products. Lane 1: molecular weight markers, lane 2–7: long range PCR products of proband. Exons amplified in each long range genomic PCR product: lane 2, exon 2; lane 3, exon 3–7; lane 4, exon 7–8; lane 5, exon 9; lane 6, exon 9–10; lane 7, exon 10. Primers details in table 1. C. Agarose gel electrophoresis of *ACVRL1* gene RT-PCR products. Lane 1: molecular weight markers, lane 2–5: RT-PCR products of proband. Exons amplified in each RT-PCR product: lane 2, exon 2–3; lane 3, exon 2–7; lane 4, exon 3–9; lane 5, exon 8–10. Primers details in table 2.

By long range PCR and sequencing assay, the genomic structure of *ACVRL1* showed that the coding region of *ACVRL1* was contained within nine exons. All except one intron followed the GT-AG rule both in proband, proband’s son and in one healthy individual. While in intron 6, displayed a nonconsensus sequence at the 5′ junction, the change gave the splice-junction sequence TAG/gcaag (Fig. 4A). The 3′ splice of this intron presented the usual consensus sequence-cag/G and the sequence result was consistent with Berg *et al.*’s report [24].

In proband, proband’s son, no additional mutations were found in the entire introns except for c.772+27G>C in intron 6. The position of each intron and the size of each exon, were determined by long range PCR amplification from genomic DNA (Fig. 4A–B, Supplementary Fig. S1).

To exclude abnormal splicing or a gross genomic rearrangement, overlapping RT-PCR was also performed and the entire coding portions of the *ACVRL1* gene were sequenced in proband, her affected son, and healthy individual (Fig. 4C, Supplementary Fig. S2). The results showed that no evidences of abnormal splice, gross genomic deletion or rearrangement were found in both HHT patients and healthy individual.

### Quantitative PCR Assays Reveal Down-regulated Expression of *ACVRL1* mRNA and Up-regulated Expression of *ENG* mRNA

**Figure 5:**
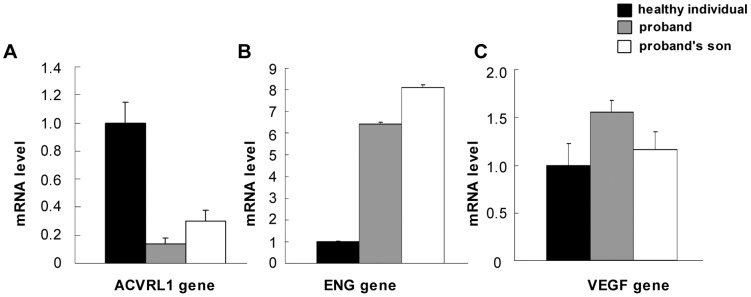
mRNA expression of the *ACVRL1*, *ENG*, and *VEGF* genes in family members with HHT. A. Expression of *ACVRL1* mRNA. The average mRNA level of healthy individuals is set to 1. The results indicate a significant decrease in *ACVRL1* mRNA levels in patients with HHT. B. Expression of *ENG* mRNA indicates a significant increase in patients with HHT. C. Assay of *VEGF* mRNA suggests a slightly increased expression in patients with HHT.

Real-time PCR was used to assess the expression levels of *ACVRL1*, *ENG*, and *VEGF* mRNA in PBMCs. The results showed that, compared with healthy individuals, mRNA level of *ACVRL1* was 6.06-fold lower in PBMCs from the proband and 2.32-fold lower in PBMCs from the proband’s son (Fig. 5A). On the other hand, the level of *ENG* mRNA was 5.43-fold higher in the proband’s PBMCs and 7.14-fold higher in her son’s PBMCs (Fig. 5B), suggesting a compensation mechanism. There also was a tendency toward upregulation in *VEGF* mRNA expression in PBMCs from the proband and her son, although the effect was not as evident (a 0.56- and 0.16-fold increase, respectively) (Fig. 5C). In PBMCs from the proband’s husband, compared with those from the healthy individuals, there was no significant difference in the level of mRNA expression of the above-mentioned genes.

### *ACVRL1* Protein Expression is Down-regulated in Affected HHT Patients

**Figure 6:**
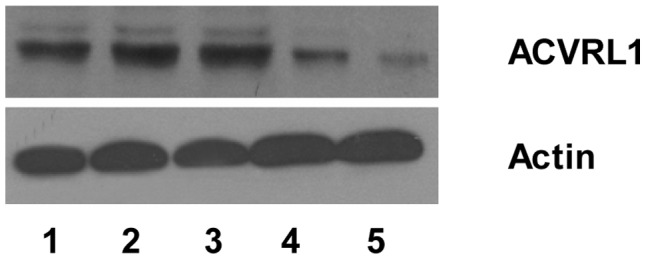
Results of Western blot analysis. Upper panel: lane 1, healthy individual; lane 2, proband’s husband; lane 3, proband’s daughter-in-law; lane 4, proband; and lane 5, proband’s son. Compared with lanes 1, 2, and 3, lanes 4 and 5 exhibit weaker ECL signals, suggesting that there is a decrease in the amount of *ACVRL1* protein. Lower panel: actin, as an internal reference.

Western blot analysis was performed to further confirm whether there was a difference in *ACVRL1* protein expression between patients with HHT and unaffected family members. The results showed that, compared with healthy family members, the proband and her affected son exhibited an obvious downregulation in the expression of *ACVRL1* protein in their PBMCs (Fig. 6).

### EMSA’s Identifies One Sp Cis-element with Decreased Affinity to the Sp1 Protein

**Figure 7:**
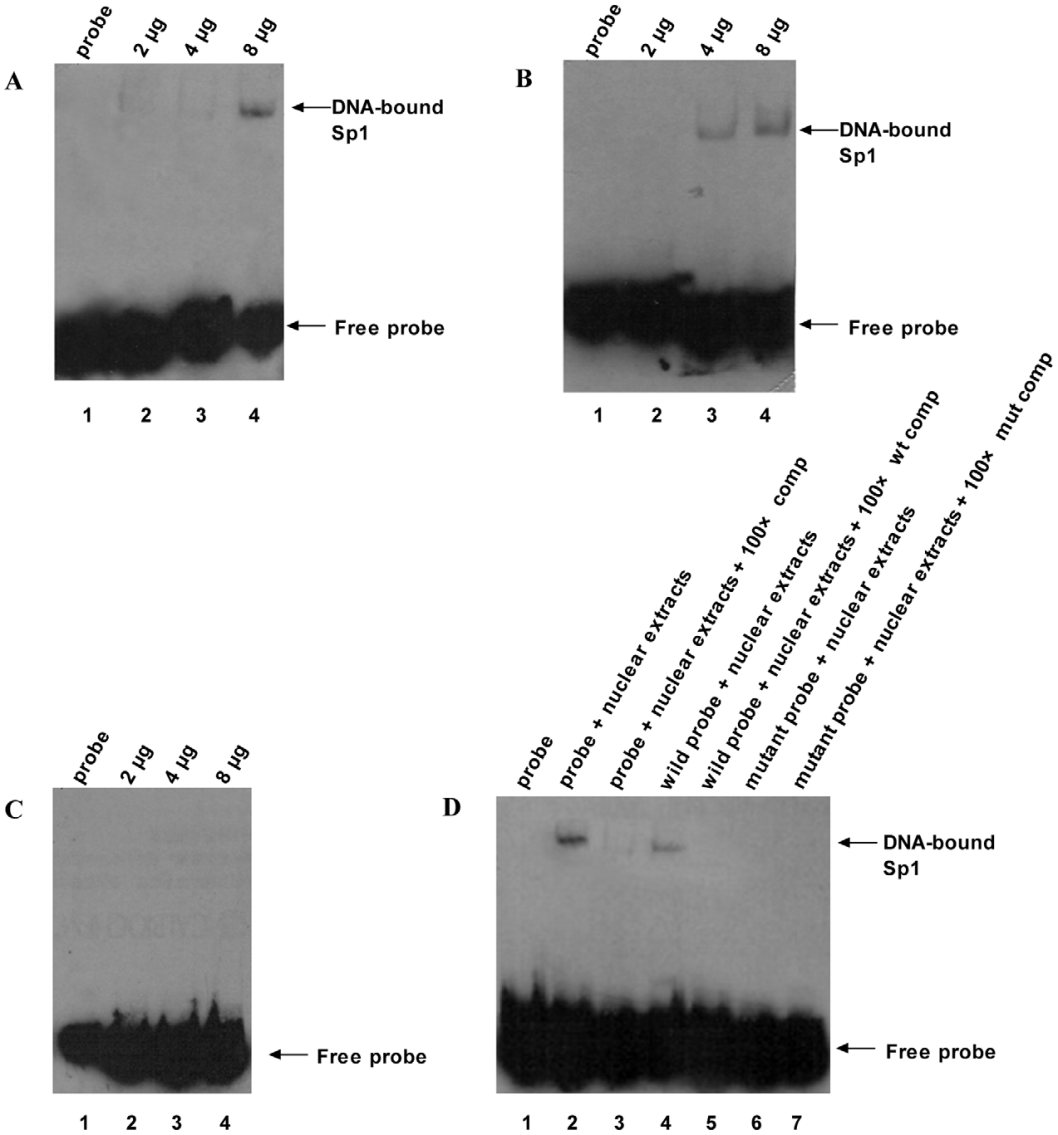
EMSA of the sequence at the intron 6. A. Biotin-labeled general wild-type Sp1 oligonucleotide probe was incubated with increasing amounts (2, 4, and 8 µg) nuclear extracts from PBMCs, and then Sp1 DNA binding activity was detected by EMSA. B. Biotin-SP1 wild-type probe, consisting of a double-stranded oligonucleotide containing the DNA sequence spanning nts +5 to +34 of intron 6, was incubated with increasing amounts (2, 4, and 8 µg) nuclear extracts from PBMCs, and then Sp1 DNA binding activity were assayed by EMSA. C. Biotin-Sp1 mutant probe, consisting of a double-stranded oligonucleotide containing c.772+27G>C mutation in intron 6 and sequence spanning nts +5 to +34, was incubated with increasing amounts (2, 4, and 8 µg) nuclear extracts from PBMCs, and SP1 DNA binding activity was detected by EMSA. D. 8 µg of nuclear extracts were incubated with biotin-labeled Sp1 probe (lane 2), wild-type probe (lane 4), and mutant probe (lane 6) in the presence of 100-fold excess of unlabeled Sp1 probe (lane 3), unlabeled wild–type probe (designated as wt comp, lane 5), and unlabeled mutant probe (designated as mut comp, lane 7), then SP1 DNA binding activity was determined by EMSA.

As shown in Figure 7, both the Sp1 general probe and the wild-type probe (a sequence spanning nts +5-+34 at intron 6) could bind nuclear extracts, and a single gel shift was observed in both cases (Fig. 7A–B). When the mutant probe was used as a target for DNA, the single band disappeared (Fig. 7C), suggesting that the mutation inhibited the binding of the transcriptional factor Sp1 in this region. In competition assays, the binding between Sp1 and nuclear extracts was inhibited by addition of excess unlabeled cold probe, and a higher mobility and weaker Sp1/DNA band were observed (Fig. 7D). Also, this result demonstrated the binding specificity for the probe/Sp1 complex.

### Reporter Gene Analysis

**Figure 8:**
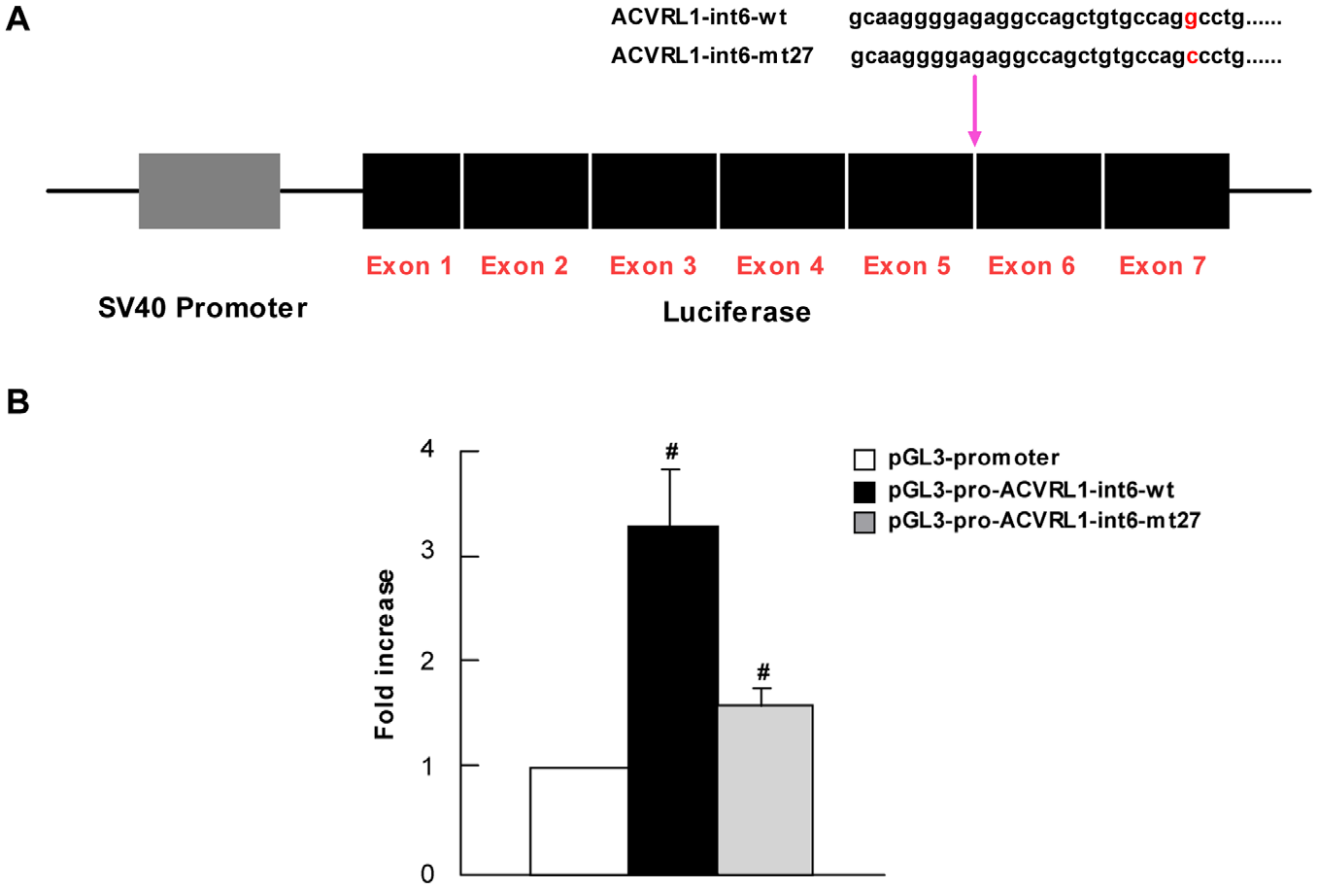
Relative luciferase activities assay. A. A schematic diagram of the reporter constructs containing the 5′ position of intron 6 from the *ACVRL1* gene. The mutant construct containing the identified mutation site (position +27) in intron 6 of *ACVRL1* from the proband is shown, and the construct was inserted into the junction area between exon 5 and exon 6 in luciferase gene. B. Results of luciferase activity were expressed as fold increases or decreases over the empty pGL3-promoter vector (^#^p<0.001). Data were from triplicate measurements with mean ± SEM.

To evaluate the effect of the G to C mutation at position +27 in intron 6 of *ACVRL1* gene, the mutant construct (pGL3-pro-ACVRL1-int6-mt27) or the wild-type construct (pGL3-pro-ACVRL1-int6-wt) (Fig. 8A) was transfected into HUVECs. Two days after transfection, the activities of the luciferase were increased 2.29-fold in HUVECs transfected with pGL3-pro-ACVRL1-int6-wt compared with the corresponding pGL3-promoter-vector transfectants (*p*<0.001). The activities of the luciferase of the construct containing the mutation at position +27 (pGL3-pro-ACVRL1-int6-mt27) were significantly decreased to 0.52 fold in transfected HUVECs, compared with the wild-type construct (*p*<0.001) (Fig. 8B). The results indicated that the mutation (c.772+27G>C in intron 6 of the *ACVRL1* gene) could influence the transcriptional activity, and might be involved in a critical enhancer element of the sixth intron in *ACVRL1* gene. Meanwhile, we found when mutant-type intron 6 was inserted into the upstream of the promoter-luc^+^ transcriptional unit of the pGL3-promoter vector, the reporter gene activity exhibited a similar decrease (data not shown).

## Discussion

We have identified a novel mutation in intron 6 of the *ACVRL1* gene. In the family study, the proband and her son displayed clinical phenotypes of HHT. The proband had gastric mucosal telangiectasias, which was identified and confirmed by magnification endoscopy with narrow band imaging (NBI). For the detection of gastric telangiectasia by the technique, few data was available. It has been shown that NBI can clearly visualize the microvascular (MV) architecture and microsurface structure, and is very useful for characterizing the mucosal vascular pattern and neoplasia in the upper gastrointestinal tract [25]. Therefore, applying NBI for the diagnosis of gastric telangiectasia may be a good choice. Some data have documented that GI involvement and bleeding occur more often in families with HHT2 [26], [27] and that the incidence of GI bleeding is 22% [28]. In our case, the proband’s gastric bleeding likely contributes significantly to her anemia. The phenotype of HHT is highly variable, even among members of the same family, and the disease displays age-related penetrance [29]. This is consistent with our observation that the proband’s son only manifested mild epistaxis, whereas the proband’s grandson, who was a “suspected” affected individual for HHT, although sharing the same mutation, had minor symptoms of bleeding.

Several papers have been published reporting the results of screening for mutations in *ENG* and *ACVRL1* genes, and more than 700 mutations have been described in these two genes [30]. As of July 2012, more than 374 mutations and polymorphisms in the *ACVRL1* gene had been recorded, which included 43 intron area polymorphisms (www.hhtmutation.org). The significance of most intron mutations is still undetermined. In the family described here, we detected a novel, previously unrecognized point mutation in intron 6 c.772+27G>C. The proband’s cDNA was normal according to direct DNA sequencing, but she showed evidence of lower amounts of protein expression in her PBMCs. Direct cDNA sequencing (*n* = 20) and StuI-RFLP (*n* = 93) of DNA samples from healthy individuals revealed no cleavage patterns, indicating that this is not a polymorphic site. More importantly, the proband’s son and grandson shared the same mutation, whereas her husband and daughter-in-law were clearly normal at this locus. The convergence of the mutation with the clinical phenotypes in the family suggests that the mutation may be associated with the pathogenesis of HHT.

In order to exclude the possible rearrangements of the *ACVRL1*gene, we amplified and sequenced the genomic structure of the *ACVRL1* gene by long range PCR, no rearrangement or intron deletion was detected in proband and her affected son. The results of overlapping RT-PCR also confirmed that the mRNA structures of the *ACVRL1* gene were integral, and no abnormal splicing was found in ours HHT patients.

The *ACVRL1* and endoglin proteins are endothelial receptors belonging to the TGF-β superfamily and are essential for vascular integrity. TGF-β signals through a heteromeric complex of type I (RI) and type II (RII) transmembrane serine/threonine kinase receptors, where *ACVRL1* is a member of the RI family of receptors and endoglin is an endothelial accessory receptor for TGF-β [31]. It is tempting to hypothesize the impact of *ACVRL1* signaling on the *ENG* promoter. Fernandez *et al.* found that mutations in the *ACVRL1* gene lead to improper upregulation of endoglin in blood outgrowth endothelial cells from patients with HHT2 [32]. Our results showed that there was a significant down-regulation in the expression of *ACVRL1* mRNA and obvious upregulation in the level of *ENG* mRNA in both the proband and her son, which is consistent with the observations of Fernandez *et al.* and suggests an imbalance between TGF-β/ACVRL1 and TGF-β/ENG in TGF-β signal transduction and a possible compensative mechanism in the *ENG* gene.

It has been shown that Sp1 is a ubiquitously expressed transcriptional factor in many organs and cells [33]. Although most Sp1 sites are found within promoter sequences, these functional sites also are present in intron sequences [34], [35], [36], [37]. More recently, Garrido-Martin *et al.* showed that Sp1 is a key regulator of the transcription of *ACVRL1* and that Sp1 binding sites are mainly located in the 5′-proximal promoter sequence of the *ACVRL1* gene [38]. In the individuals with HHT that are presented here, the proband’s cDNA sequence was normal, but she expressed lower amounts of the *ACVRL1* protein in her PBMCs (Fig. 6), suggesting that the point mutation in intron 6 influenced the expression of *ACVRL1* protein through the down-regulation of gene transcription. The result is consistent with the lower level of expression of *ACVRL1* mRNA (Fig. 5). Additionally, there was no splicing defect in the exon-intron boundaries of the proband’s *ACVRL1* gene. We hypothesize that the nucleotide mutation affects a critical enhancer element in the sixth intron and infer if the mutation affect transcriptional activity of the *ACVRL1* gene. Our results show that the nucleotide sequence of intron 6 in the *ACVRL1* gene greatly influences Sp1 binding, and that the presence of the G nucleotide is necessary for Sp1 binding activities. It is possible that Sp1 or its binding sites are involved in the basal activation of the transcription of *ACVRL1*. In addition, we found that the transcriptional activities produced by pGL3-pro-ACVRL1-int6-mt27 in HUVECs were significantly lower than the wild-type construct. Therefore, our results confirm that the nucleotide region at position +27 in intron 6 presents a possible positive regulatory element for controlling the basal transcriptional activity of the *ACVRL1* gene and the mutation at this site may influence promoter activity.

Overall, this study provides a detailed analysis of a Chinese family with HHT2. The study included description of the clinical features, documentation of the extent of telangiectasia, and identification of a new mutation in the *ACVRL1* gene. We also analyzed the alterations in the transcriptional levels that may be associated with the mutation in intron 6 and found that the mutation has functional consequences. EMSA indicated that intron 6 c.772+27G>C plays a role in Sp1 binding, suggesting that the region is part of a novel transacting element that may be involved in the transcriptional regulation of the *ACVRL1* gene. The identification of a novel point mutation in the *ACVRL1* gene may be important as a molecular diagnostic marker and elucidating the pathogenesis of HHT.

## Supporting Information

Figure S1 **The sequencing result of** ***ACVRL1*** **gene by long range PCR.** Genomic DNA was used for amplification of *ACVRL1* gene, sequence results are as below (proband). Double underline = primer sequence (details in table 1), black = intron sequence, red = exon sequence, purple = c.772+27G>C mutation.
(DOC)

Figure S2 **The coding sequencing result of** ***ACVRL1*** **gene by RT-PCR.** Overlapping PCR was carried out for amplification of *ACVRL1* cDNA, sequence results are as below (proband), double underline = primer sequence (details in table 2).
(DOC)
