# Supplementary material for: An Intron Mutation in the ACVRL1 May Be Associated with a Transcriptional Regulation Defect in a Chinese Family with Hereditary Hemorrhagic Telangiectasia
Source: PLoS One. 2013 Feb 27;8(2):e58031. doi: 10.1371/journal.pone.0058031 (PMC3584037; doi:10.1371/journal.pone.0058031)
Supplement: Figure S1 — The sequencing result of ACVRL1 gene by long range PCR. Genomic DNA was used for amplification of ACVRL1 gene, sequence results are as below (proband). Double underline = primer sequence (details in table 1), black = intron sequence, red = exon sequence, purple = c.772+27G>C mutation. (DOC) [file pone.0058031.s001.doc]

**Figure S1. The sequencing result of *ACVRL1* gene by long range PCR.**

agcaaaggctgccaagga^1-sense^tggcttcctgctagtaccccagtgggcagattcagctcctctagggtgctctctccatcccatcccaccgtcagtcctgccgcacagtgctagccagagtcctcactggctgcgggaggggcaagaggcaggaggtcaggcagaggctggggacacagctgcagcagcccagggctctgtttcctcccccagcctgggcctctggggcagaggaagcccaggcaggaaggccaggcctgccaccagccagagaggcctatgaagcccccctaccccacccctagccatactgccctcagagcctcagcatcttgggtcttcatttggcaccattcttagactatgggctttctgaagcctctgtggccatgtgcccagccctgtcaggctcaccatgtcatgccaaaggacagtctccctgccttgtgccctgcctgaggatccagtccccagacacacacctgcacatgctccaccattcagaaagtcctgttgctgtctgaccagggtccttcttgctgcagctttagccaatatcttctggttctaaacctgtgagaaatgaagactgggtggcttcagaagcactagttggtgtgggacctggattggggactatcatagaccagaattactccccctaacactctccaacaaacttctggaaaagttcaaccctagtcagccatggaaggtcaaacattcagtgtctaagtgtgtttcacttcccctgccacatcactggccctccacccaccctttcctagatggcaaggggaaagaagggggccagggaacagctccgagagggcagcttatactctggtgagcttgcagagaggtttctctacacctggcatgaccctgccccgtccgagctttgcatggttgttatagccctgagggctcagacatgggctgccaccactggatagggacagagcgaagagagcacttgattaggagtctggaaacctgggatgtacgcctgctggtgccagtgagtcctgtctgacccgggacaagtcttcttccctgtatgagccttactcctctcttctgtaaaacaaggagctggattttgtgattcacaggatgtccacctagaattctgtcatgtgggtgtcccaggcacaaggaactaagggtagcaaggaactaagggcatatttaaaaagcccctccagaccttgtggctgctgcagcccaccctgaaaatatgccctcagttccctgctgtgcgtgactcctgcctgttctatccagaccccaatctaaacaatcttgattcctgttcctggcttggcaggaccctgaatggcaggaagcgaagacaggagcctgtttatgtttgaggcagccagggctgggggggcattgagaaagggtaggcaggggtggaagctgtgagggagtgggaaagagacacagaacagaaggggagctgctggagagatgctccttctaccccaattgggtgctccctgcggctctcccaacctgcctgcagtctgagctcagcagcaggagtgcagagctagggtttccccaagctctcagtcacttaaacattgctctccacccttcacctctaacaggatggtttccatggggaagtgaaccaggacttcccctgcaggccccgccccaaagccaggcggcagggagtagggaggcggcctccctgcctcccctccaaaaaaaactctgtgatttcctctgggcaggagggagccacggccagcggctgtcacacttcatggctcttactccacctctcttgctcctctctgcagggaccatgaccttgggctcccccaggaaaggccttctgatgctgctgatggccttggtgacccagggtgagtactgggggagcagttaggaaacagga^2-sense^acctggatacagaaagggctatctgggcccagatcagctctgcctggggctgaacttgagaagctggggagaatgtaggagcttgactggagagtggaggacagtgaggctccattagactcagtccccagctaccccagccctcctttgctctcctcttgatccagacctgcaggctggagctctgtcagactagggtggaagcctatatgtggggtggagggagagcaggtgttggcaggccaagcctgggagagcactcagggctggggctgggggcccagcccagcctagaactgggaggggaggacctaggagccaggaggggacaaaagcctgcttttcagagtgtcagctgctctgaggggagggttctgagggaaggatgactgaggatgaaagtaagagaccaaagcttcaaggtgtttgtctgaggggtcagacgagagggacagtaggacagaaatgggtgtcgggctcag^1-antisense^cctgggggagctgggaccacagtggctgagcttccggtgtgtcttccaggagaccctgtgaagccgtctcggggcccgctggtgacctgcacgtgtgagagcccacattgcaaggggcctacctgccggggggcctggtgcacagtagtgctggtgcgggaggaggggaggcacccccaggaacatcggggctgcgggaacttgcacagggagctctgcagggggcgccccaccgagttcgtcaaccactactgctgcgacagccacctctgcaaccacaacgtgtccctggtgctggagggtacgtccagctgccctagcactccctccccatcttcttggcccctgccctcccttccctcctttcctctcatgctctggccaataaaggggctgggggcgggggagcgggtgggcaggactctgggatctaactggcagagtggtctggcccgaggtggggggagctgacctagtggaagctgagcctcagtgtccccctccctcagccacccaacctccttcggagcagccgggaacagatggccagctggccctgatcctgggccccgtgctggccttgctggccctggtggccctgggtgtcctgggcctgtggcatgtccgacggaggcaggagaagcagcgtggcctgcacagcgagctgggagagtccagtctcatcctgaaagcatctgagcagggcgacagcatgttgggggtatgggcctggggacctgggacacagggtgtaggaggggcagataggaactgcagaatcagaggggtcacccagagattagagccggtggggagctgggcgagtgaggagcttgcagtgacccagcaggtcccaggtcgaggatagagaagggggctgtggctggttgtggcagcctctcagtggcctctccgtacccccaggacctcctggacagtgactgcaccacagggagtggctcagggctccccttcctggtgcagaggacagtggcacggcaggttgccttggtggagtgtgtgggtgagcagtgggtgagcccggtggatgaggaccaagggctctcatgagcctggaggggtgagggagtttttggctactggaatcacaggcggtgccaggcctgggtcagaattggaattctgctgggcagggagtgggctggagacgggccagggctaggttcttctttctgcaggaccggggtggaacgagaggcagctgggggtggcctgccactgggtttgggtctggattaagttaaacctaagggtctggggttctgtgggtggggtgggcgagggaggcagcgcagcatcaagatggggggctcttccagggctctgtgtgcccagtgtgtaaccctcaccttcccctctggccatcaggaaaaggccgctatggcgaagtgtggcggggcttgtggcacggtgagagtgtggccgtcaagatcttctcctcgagggatgaacagtcctggttccgggagactgagatctataacacagtgttgctcagacacgacaacatcctaggcaaggggagaggccagctgtgccag**c**cctggggctttgcccccctgcactcagggctcaagtttgcagacctccagacattaacagaaccctgaaggactctcagcccacctgcagcatcaacctctttttttaatttaatttaatttaatttaatttaatttaatttaatttaatttttgagacagggcctcgctctgtcacccaggctgggatgcagtggtgcaatctcactggagcctcaatctcttgagctcaagtgatcctcccacttcagcctcttgagtagctgggaccacaggcatgtgccaccatgcccagctaacttttttattttttgtagagacgaggtctccctatgt^3-sense^ttcccagactggtctcaaattcctaggctcaagcagtgctcttgccttggcctcccaaagtgttgggattacaggtgcaagccactaagccaggcccagacccaacctctgactccagctctgtctctgacctaagccacatcaacccccacccccagacctagcttagcagtgacccagtccattccctctcccccaaccccaccctgaccctgacgactccagcctcccttagccccagccccttggctgagtcacccaacctttctgcacacaggcttcatcgcctcagacatgacctcccgcaactcgagcacgcagctgtggctcatcacgcactaccacgagc^2-antisense^acggctccctctacgactttctgcagagacagacgctggagccccatctggctctgaggctagctgtgtccgcggcatgcggcctggcgcacctgcacgtggagatcttcggtacacagggcaaaccagccattgcccaccgcgacttcaagagccgcaatgtgctggtcaagagcaacctgcagtgttgcatcgccgacctgggtgagccgggcggggcaggggcgcgcccttcacaggtgggcggagcttgtgcgctctcctctcctttgcctgtgggcggtgaccatgattagcacttgaaaatttagaggtgcatgttgtttcagttctcctccgcaagaccccacgagttgtctgaacacccttttcaaacccagattccttccaccataccatcctggctccaggagttgggagaaaggaggcaggagccaggaacctgggttctcatcctggtttcgccagcctccaggtctgctctgtgaagtgggctcataatgcctgccttgcccactcacatctcgctgggttcttgggaacctagagagcactacacaagcaaagagtacctggggccatggttctctctgtggccactgccttccagcccatctccgtgcacgtctccatctgccttcccctctctgtcccactgtttctctcagtccccaccttgcctgccccctggatcccaggtttgggagaggggcaggagtgacaggcctcacccccacaggcctggctgtgatgcactcacagggcagcgattacctggacatcggcaacaacccgagagtgggcaccaagcggtacatggcacccgaggtgctggacgagcagatccgcacggactgctttgagtcctacaagtggactgacatctgggcctttggcctggtgctgtgggagattgcccgccggaccatcgtgaatggtgagggcccaccctacacagggtagggaaaggggaatcagcctgtggagccaggggcttccagccatggccagtgcccatggcctgggaggtttgcagtcagacctcctggcaccccttccatgctgcccaccagctggttcagctgagtgaccttttaaggtataaaccttaaaaacagataacagggtctgtgagtctggctgtgaaatcttgggtacatgataataacagaaatattaatagcagctaatattgattgagcttaaatatgtgccagatgccatgctatgcactttacatgtattatttaattctttggtatggacaaggcagtggtattgcttagtggctaagagcataggcttggaatcagacttcctgggttctattcctgatcttgccatgaccgtgcgtaggttatttgac^4-sense^ctctctgtgcctcagcttcctcatttgtaaaatgggtgcgatggctcatgcctgtaatcccagcactttgggaggccgaggcaggcagatcacctgaggtcaggaattcaagaccagcctggccaacatggcgaaaccctgtctgtactaaaaatacaaaaattagctggctgtggtggcacacacctgtaatcccagctactcgggaggctgaggcaggagaatcacttgaatctaggaggcagaggttgcggtgagccaagatcatgccactgcactccagtctgggcgacagagtgagaccctgtctcaaaaaaagaaaatttattaaaataaataaataaaatgagaataaaaatagcttccagttgttgagataatctaagtatttagaacagtatctagagctaaatattagtggctgttattgttgtagatagaatgagtgcctgacatcatattgtccccattttacagttgaggagacgggggcacagaatgacagtgggcttgaggcagcatcagggtccgaaaccgggcagtctgccccggggccagtgctcatcatcactgtgtgcacttaaacctctctggcccttgatttcctcatgcacgcaatgcatgtgagtgcctgcactgcctgcttattgctgcctggttgttactgtgggttgc^3-antisense^cacaggggactctgatttagagggactgcgacaggtagagagacctgccctgggcaggcagccctgaggtcgatgttctctcagccctggagtggacggaggataggtgggtcgtctagactggtgggagcattgtcaacctttgaggaggctgtccatggtgagggacttccaggagtcgtgacaggttggggacaatcctcaggatatggctggagccctgcttcttggggacaaggatgtcctcatttcctgagcatctaccaggagccagccccatgccagacttcatttgtcctcggtggtcatcaactgaaaacagaggctgtggtgtcaccggtcccttggggagactcacgaggtgcttagattcctcaagactccgggcatgaattgccagagtgggctctaaggagaccgggatcgggggaaagaggaacctggaagaagccaaggctccttggaccaggctgcagggcagaaaggaaaggctgggaggaggctgcctggggtggagggggaagctccatcagccccacacggactcgcggcgcattataaacactgtaatctggtgtcagccccggcactgattaaaggcccattagacacattcaggcctctgtgcagcactgattagggcgtgagcggcacaggggccggcctggaagctggccatggggagaacagctggcgaaggctctatcgaggttgcggcctgtgtggccatggcccccagcctcaggctgggaaacaggatggggcacggggctgtctgcggctccaccggcattgttttcagaaagctcctactaaaaactgtgaacttgggataccaagaggagggggtcagtgctggctgcaggagtcagcagaggcctgtgccatccctgacactgggtgatgttatgtggacctgggtaaaaagataagtgcagctcagaggtcaagagcacatgctttggtgttagactgtgggcttaaatcctagccctgtcacttactatctgttgatcttagacaagtttcttaaactctctggccttcatttcttggcttataaagtggagctcatagtattacctacctcatagggctgctgtgaggatttcatgcagagctcttaggacagcacctggcatacagtgctcagtaaacatttggtgttattattatcaggcatgtgtgatagttggggcctgggagcagtcccgggcactgggtggaaggcagctgagggaagactggaacaaggctggcctaggaatccgagatgggtttgagtcttgcctctgccattactagctgtggaaccttgggccccccttctctgagcatcagtttcttcatctgtaaaatggaggtaatactagtacccaccccacagggctgttgtgagga^5-sense^caaacaggataaagcatgtaaagtgtttacagcagaatatctggcacatgccatgtgcaaaacaaatggcagtcattaacattagttagaaacacatttattgcattatactgtccctctcaggggtagcgtgtccaggccactggtttctggcccttggatagagggtagaaaaggctctcctctgggtggtattgggcctccttagagtcccaagtgattgtcctgtccattctccatttccaggcatcgtggaggactatagaccacccttctatgatgtggtgcccaatgacc^4-antisense^ccagctttgaggacatgaagaaggtggtgtgtgtggatcagcagacccccaccatccctaaccggctggctgcagacccggtgaggcctctgctgggactaggatggcgtggggtggtggctcatggctgggatttctgggcccaggaacttgtgtctgaggcctctgcttcatctcatagatactgagtgtcctggttagggcacctctctaggtactccctctttcaaccctggcccatgctccctgcccccagggccatctggtcattctgggattttaagaaccttcaccttccctgtgaaaagtcagaggctatctgtggctctgtgtgtgtgtgtgtgtgtgtgtgtgtgtgtgtgtgtgtgtttgagagtcagggttttgtttgctcttgtcacccaggctggggtgcagtggcacagtcatagctcactgtaacctcaaactcctcggctcaactgatcctactgcctcagcctccccagtagctaggtgtagtgcacaccaccacacctggctaatttttaaaattttttgtagagacaaggtcttttatgttgctcaggcttatctcaaactcttgggctcaagtgatcctcctgcctcagcctcccaaagtgttgggattataggtgtgagcctccacacctgaccactgtggtatttttgttgctatttgtctttgtttgttgctgttttgttgttgtttgtccttgttttttctcaaaacacttcacttgtacaaagacacacacacgtatatcaacacatacaagcgtgaatatccttctgaacatacactcacagaaatatacacatgcgcatatatgccagactgttggccccgtgaggacaaagaacgtgtcttttgctcacccttatgtccctattacctatcatagtatctggctcacaataggtgctaataaatatttactgaaagaatgaatatattctatacatgtatacatctatgtgtacatagacatacatttctgtacaacatatccgcaaaaagccacatgtatgcattcctgtacacacacacatacactcactttcaggagccagaagtggggccagctcatttcaggccctgggatgggttgatgagcttcctgggatgggttgatgagctttttgggatgggttgctccctggacacttgcatttttagggcagttcccacttccacagtcccctgggcccagccatggctgatggaggggctggaagacactgccctctgcactggggtcttaggctgtggatggggatgtgggagcccccaggcttgaagaatcggccccttcctatggatcttggcaaaggtggggatgggggtaaggtgtttcacagacactgcttgcccaagggggcttgagtgctcctgaagatctgagttctctctttctgtccgccctgtctttctccatccctttctcactctactctcttcctcttccattttttatttttttgtcatcctctgttctctctctcaccacttgtctttcctctcgctctcctttccaatgctctcatctccctcctctcatcctttctctcctgcttatgtctccccattaccggccatcctcctcatcttcttcccatcttctctgacccacctccctctgcatctctctcccgaccccctcctcttctctgcatctctctctctgcctcctctcctctgcacctctctcccaacccccaggtcctctcaggcctagctcagatgatgcgggagtgctggta^6-sense^cccaaacccctctgcccgactcaccgcgctgcggatcaagaagacactacaaaaaattagcaacagtccagagaagcctaaagtgattcaatagcccaggagcacctgattcctttctgcctgcagggggctgggggggtggggggcagtggatggtgccctatctgggtagaggtagtgtgagtgtggtgtgtgctggggatgggcagctgcgcctgcctgctcggcccccagcccacccagccaaaaatacagctgggctgaaacctgatcccctgctgtctggcctgctcaaagcggcaggctccctgacgcctggctctctccccacccctatggccagcatggtgcaccccctaccactcccgggacaggatgcaaaagaggctccagagtcagagtgccaagccagggaa^5-antisense^tcccagtcccagactcagagcccgggcctgcactttgccccctgcccttgatcaaccccactgccccaccagagctgccagggtggcacagggccctgtccagcccctggcacacacttccctgccaggcctcagcctctagcataagctccagagagccagggcccatcagtttctctctgtggatttgtatctcagctccatgatgccttgggctttctgtctcctcaacaagagtgcagcttgctgaatgtcagctgcctgagagagctggggcctgacttactagggcattaaatcctaagaggtcctactgaggtgtggcaggatcacaggccagtggaaaaagggcaggtcagatgggcaaggcccaggactttcagattaactgagaggatatcgaggccaagcatggcagggggaaggtcagtgggtgtcaagagacccaggtctgaccccggatgtttgctccatgtgacaaaagcaggcctgtctcaggaccttttcttttcttttttccttctttttttttttgacacggagtttcgctcttgttgtccaggctagagtgcaatggcatgatcccagctcaccgcaacgtctacctcccaggttcaaatcattctcttgcctcagactcccgagtagctgggattacaggcacatgccaccatgcctggctaattttgtatatttagtagaaacagggtttcaccatgctggccatgctggtctcgaactcctgacctcaggtgttccacctacctcagcctcccaaagtgctggggttacaggtgtgagccatcgcgcctggccaggacctttgtttcttatctacatattggaagatttggtcctgatgtcctttgaggcttctttagctctagttctctgacacttcagcctatatcacagctaacttcttcagtctcatctattccttatgctccagcccctggcaatttgcctcaagatgggggtttgaaaataactttacctgactcaaggagtgtctggagcacctcctagtctaagtctgcaagctccagttcttgcctaaaaccatgccagtggccacccttgggctcagacagctctgggccttttgaccacaagccagcccctcgccctctctgtggcatagtcttctctgccccaggactgcagggcggcttcctccaaggcttccaaggctcaaaagaaatttggctccatccaagaaggctccagctcccctactggcccctggctcaggcccacacccctggccaggcccagagagtgtgtctcaggagaattcaatggctctagagagacacacagaaagtttggcatttggaaatttcaaggatgtatgtatgctcacgtatggagcaggttgtcctggtccctgggtgcagggaagtgggctgcagggaagtggattggaggggagcttgaggaatataaggagcgggggtggagactcaggctatggacaaggacagccccaaggttgggaagacctggccttagtcgtcctcagcctaggggcagggcagtgaagaaagctctccccgctcctgctgtaatgacccagagtagcctccccaggccggcatcttatgtgtgtcttccaccatcctcatggtggcacttttctaggcctgtctcccagcattgtgcaaggctcggaagagaaccaggaagtgaaactgggtgaaaacagaaagctcaatggatgggctaggttcccagatcattagggcagagtttgcacgtcctctggtcactggaatccacccagcccacgaatcatctccctcttgaaggattttatttctactgggttttggaacaaactcctgctgagaccccacagccagaaactgaaagcagcagctccccaaagcctggaaaatccctaagagaaggcctggggcaggaagtggagtgacaggggacaggtagagagaagggggcccaatggccagggagtgaaggaggtggcgttgctgagagcagtctgcacatgcttctgtctgagtgcaggaaggtgttccagggtcgaaattacacttctcgtacctggagacgctgtttgtgggagcactgggctcatgcctggcacacaataggtctgcaataaaccatggttaaatcctgaagctccggagtcttcccaggccgatgcctctgcagtggcccgtcgcagaggtccagataaaaccagagggatccggggagaagagggtcccaggatacacgcccagctttgcccttagacaggtcacagtttcctcacgcccctgctcagacccccgcgggggcccaaatctcagttcctaccccagattggga^6-antisense^
