# Supplementary material for: An Intron Mutation in the ACVRL1 May Be Associated with a Transcriptional Regulation Defect in a Chinese Family with Hereditary Hemorrhagic Telangiectasia
Source: PLoS One. 2013 Feb 27;8(2):e58031. doi: 10.1371/journal.pone.0058031 (PMC3584037; doi:10.1371/journal.pone.0058031)
Supplement: Figure S2 — The coding sequencing result of ACVRL1 gene by RT-PCR. Overlapping PCR was carried out for amplification of ACVRL1 cDNA, sequence results are as below (proband), double underline = primer sequence (details in table 2). (DOC) [file pone.0058031.s002.doc]

**Figure S2. The coding sequencing result of *ACVRL1* gene by RT-PCR.**

Amplification of *ACVRL1* cDNA by using primer #1:

tgtcacacttcatggctcttactc^1-sense^cacctctcttgctcctctctgcagggaccatgaccttgggctcccccaggaaaggccttctgatgctgctgatggccttggtgacccagggagaccctgtgaagccgtctcggggcccgctggtgacctgcacgtgtgagagcccacattgcaaggggcctacctgccggggggcctggtgcacagtagtgctggtgcgggaggaggggaggcacccccaggaacatcggggctgcgggaacttgcacagggagctctgcagggggcgccccaccgagttcgtcaaccactactgctgcgaca^1-antisense^

Amplification of *ACVRL1* cDNA by using primer #2:

cttcatggctcttactccacctc^2-sense^tcttgctcctctctgcagggaccatgaccttgggctcccccaggaaaggccttctgatgctgctgatggccttggtgacccagggagaccctgtgaagccgtctcggggcccgctggtgacctgcacgtgtgagagcccacattgcaaggggcctacctgccggggggcctggtgcacagtagtgctggtgcgggaggaggggaggcacccccaggaacatcggggctgcgggaacttgcacagggagctctgcagggggcgccccaccgagttcgtcaaccactactgctgcgacagccacctctgcaaccacaacgtgtccctggtgctggaggccacccaacctccttcggagcagccgggaacagatggccagctggccctgatcctgggccccgtgctggccttgctggccctggtggccctgggtgtcctgggcctgtggcatgtccgacggaggcaggagaagcagcgtggcctgcacagcgagctgggagagtccagtctcatcctgaaagcatctgagcagggcgacagcatgttgggggacctcctggacagtgactgcaccacagggagtggctcagggctccccttcctggtgcagaggacagtggcacggcaggttgccttggtggagtgtgtgggaaaaggccgctatggcgaagtgtggcggggcttgtggcacggtgagagtgtggccgtcaagatcttctcctcgagggatgaacagtcctggttccgggagactgagatctataacacagtgttgctcagacacgacaacatcctaggcttcatcgcctcagacatgacctcccgcaactcgagcacgcagctgtggctcatcacgcactaccacgagcac^2-antisense^

Amplification of *ACVRL1* cDNA by using primer #3:

cgtcaaccactactgctgcga^3-sense^cagccacctctgcaaccacaacgtgtccctggtgctggaggccacccaacctccttcggagcagccgggaacagatggccagctggccctgatcctgggccccgtgctggccttgctggccctggtggccctgggtgtcctgggcctgtggcatgtccgacggaggcaggagaagcagcgtggcctgcacagcgagctgggagagtccagtctcatcctgaaagcatctgagcagggcgacagcatgttgggggacctcctggacagtgactgcaccacagggagtggctcagggctccccttcctggtgcagaggacagtggcacggcaggttgccttggtggagtgtgtgggaaaaggccgctatggcgaagtgtggcggggcttgtggcacggtgagagtgtggccgtcaagatcttctcctcgagggatgaacagtcctggttccgggagactgagatctataacacagtgttgctcagacacgacaacatcctaggcttcatcgcctcagacatgacctcccgcaactcgagcacgcagctgtggctcatcacgcactaccacgagcacggctccctctacgactttctgcagagacagacgctggagccccatctggctctgaggctagctgtgtccgcggcatgcggcctggcgcacctgcacgtggagatcttcggtacacagggcaaaccagccattgcccaccgcgacttcaagagccgcaatgtgctggtcaagagcaacctgcagtgttgcatcgccgacctgggcctggctgtgatgcactcacagggcagcgattacctggacatcggcaacaacccgagagtgggcaccaagcggtacatggcacccgaggtgctggacgagcagatccgcacggactgctttgagtcctacaagtggactgacatctgggcctttggcctggtgctgtgggagattgcccgccggaccatcgtgaatggcatcgtggagga^3-antisense^

Amplification of *ACVRL1* cDNA by using primer #4:

gattacctggacatcggcaacaac^4-sense^ccgagagtgggcaccaagcggtacatggcacccgaggtgctggacgagcagatccgcacggactgctttgagtcctacaagtggactgacatctgggcctttggcctggtgctgtgggagattgcccgccggaccatcgtgaatggcatcgtggaggactatagaccacccttctatgatgtggtgcccaatgaccccagctttgaggacatgaagaaggtggtgtgtgtggatcagcagacccccaccatccctaaccggctggctgcagacccggtcctctcaggcctagctcagatgatgcgggagtgctggtacccaaacccctctgcccgactcaccgcgctgcggatcaagaagacactacaaaaaattagcaacagtccagagaagcctaaagtgattcaatagcccaggagcacctgattcctttctgcctgcagggggctgggggggtggggggcagtggatggtgccctatctgggtagaggtagtgtgagtgtggtg^4-antisense^
